# Amoebal Endosymbiont *Protochlamydia* Induces Apoptosis to Human Immortal HEp-2 Cells

**DOI:** 10.1371/journal.pone.0030270

**Published:** 2012-01-19

**Authors:** Atsushi Ito, Junji Matsuo, Shinji Nakamura, Asahi Yoshida, Miho Okude, Yasuhiro Hayashi, Haruna Sakai, Mitsutaka Yoshida, Kaori Takahashi, Hiroyuki Yamaguchi

**Affiliations:** 1 Department of Medical Laboratory Science, Faculty of Health Sciences, Hokkaido University, Sapporo, Hokkaido, Japan; 2 Division of Biomedical Imaging Research, Juntendo University Graduate School of Medicine, Tokyo, Japan; 3 Division of Ultrastructural Research, Juntendo University Graduate School of Medicine, Tokyo, Japan; University Freiburg, Germany

## Abstract

*Protochlamydia*, an environmental chlamydia and obligate amoebal endosymbiotic bacterium, evolved to survive within protist hosts, such as *Acanthamobae*, 700 million years ago. However, these bacteria do not live in vertebrates, including humans. This raises the possibility that interactions between *Protochlamydia* and human cells could induce a novel cytopathic effect, leading to new insights into host-parasite relationships. Therefore, we studied the effect of *Protochlamydia* on the survival of human immortal cell line, HEp-2 cells and primary peripheral blood mononuclear cells (PBMC). Using mainly 4′,6-diamidino-2-phenylindole staining, fluorescent *in situ* hybridization, transmission electron microscopy, and also TUNEL and Transwell assays, we demonstrated that the *Protochlamydia* induced apoptosis in HEp-2 cells. The attachment of viable bacterial cells, but not an increase of bacterial infectious progenies within the cells, was required for the apoptosis. Other chlamydiae [*Parachlamydia acanthamoebae* and *Chlamydia trachomatis* (serovars D and L2)] did not induce the same phenomena, indicating that the observed apoptosis may be specific to the *Protochlamydia*. Furthermore, the bacteria had no effect on the survival of primary PBMCs collected from five volunteers, regardless of activation. We concluded that *Protochlamydia* induces apoptosis in human-immortal HEp-2 cells and that this endosymbiont could potentially be used as a biological tool for the elucidation of novel host-parasite relationships.

## Introduction

Chlamydiae were once considered a group of closely related bacteria comprising many important human and animal pathogens that have a recurrent developmental cycle between the elementary body (EB) and reticulate body (RB) forms in inclusion body surrounding by membrane vesicle [1], [2]. These bacteria, now so-called pathogenic chlamydiae, can cause a variety of diseases such as pneumoniae, trachoma, and urogenital tract infections, which are a major cause of female infertility [3]. *Chlamydia trachomatis* is the most frequently sexually transmitted bacterial pathogen worldwide, with over 90 million new cases of infection per year [4], [5]. *Chlamydophila pneumoniae* is implicated in several chronic diseases, including atherosclerosis [6] and central nervous system diseases such as Alzheimer's disease and multiple sclerosis [7]. All of the pathogenic chlamydiae species have co-evolved with their vertebrate hosts, including humans, over the past 700 million years. Such relationships between these bacteria and their hosts are therefore postulated to be symbiotic. Stable and exclusive host-parasite relationships have developed through a decrease in genome size and loss of redundant genes, resulting in a shift to parasitic energy and metabolic requirements and genomes of approximately 1.0–1.2 Mb [8], [9], [10]. This gene reduction is potentially a strategy to efficiently escape from the host-immune network [8], [9], [10]. The complicated mechanism between pathogenic chlamydiae and host cells are becoming more obvious. It is the striking view that chlamydial type III effector proteins, inclusion membrane proteins (Incs) are deeply responsible for a process of inclusion biogenesis [11]. Furthermore, while the pathogenic chlamydiae also possess chlamydia protease-like activating factor (CPAF) causing modification of cellular function [12] and actin-recruiting protein Tarp [13], it is a notable thing that these bacteria have chlamydia protein associating with death domains, inducing apoptosis in variety of mammalian cell lines [14].

Several chlamydia-like endosymbionts of amoebae, inhabiting environments including pond water, soil and sewage, were discovered in the late 1990s, demonstrating the existence of a broad range of chlamydiae in diverse natural habitats [15], [16]. Numerous new chlamydial organisms, including the so-called environmental chlamydiae, have now been assigned to the new families *Parachlamydiaceae*, *Simkaniaceae* and *Waddliaceae* [17]. While these environmental chlamydiae have a developmental cycle similar to the pathogenic chlamydiae, they differ in that they evolved to live with a protist host, such as amoebae, some 700 million years ago [10]. In addition, the genome of environmental chlamydia *Protochlamydia* UWE25 is not in the process of becoming smaller and has stabilized at 2.4 Mb [10]. This implies that to overcome stressful conditions, the environmental chlamydiae still possess certain molecules that the pathogenic chlamydiae have lost. It is also possible that interactions between these bacteria and human cells could induce novel cytopathic effects, leading to new insights into host-parasite relationships.

The family *Parachlamydiaceae*, consisting of *Parachlamydia acanthamoebae*, *Neochlamydia hartmanellae* and *Protochlamydia amoebophila* has well documented as representative environmental chlamydiae that show a wide distribution range in natural environments, such as in rivers and soil [10], [15], [17]. In particular, it is well known that *P. acanthamoebae* infects and to a limited degree multiplies in human cell lines such as monocyte-derived macrophages, pneumocytes, and lung fibroblasts [18], [19], [20], and the bacteria has been implicated primarily in community-aquired pneumonia, bronchitis, and aspiration pneumoniae [21], [22]. Recently, it has also been reported that *Protochlamydia* is a potential etiological agent of human pneumoniae [23], although the association of the *Protochlamydia* with human cells possibly causing cellular function such as cell death remains unknown.

We previously isolated five *Acanthamoeba* strains from river and soil samples in Sapporo, Japan, which were persistently infected with the endosymbiotic bacteria α-*Proteobacteria* and β-*Proteobacteria*, and environmental chlamydiae including *Neochlamydia* and *Protochlamydia* [24], [12]. Our data indicated that the viability of all endosymbionts isolated from the amoebae rapidly decreased after 10 to 24 hours and lost the ability to be transferred to other amoebae strains [24], [25]. This indicated a stable symbiotic relationship between the host amoebae and these bacterial species. As mentioned above, *Protochlamydia* is likely to have retained many fuctional molecules, which pathogenic chlamydiae have already lost as a consequence of adaptation to a stable host environment. In the present study, we therefore assessed the potential induction of apoptosis by the amoebal endosymbiont *Protochlamydia* on the human immortal cell lines, HEp-2, and primary peripheral blood mononuclear cells (PBMC), offering new insight into host-parasite relationships and the development of novel strategy for removing cells persistent infected with pathogens.

## Results

### Effect of bacterial treatment on human immortal HEp-2 cell morphology

**Figure 1:**
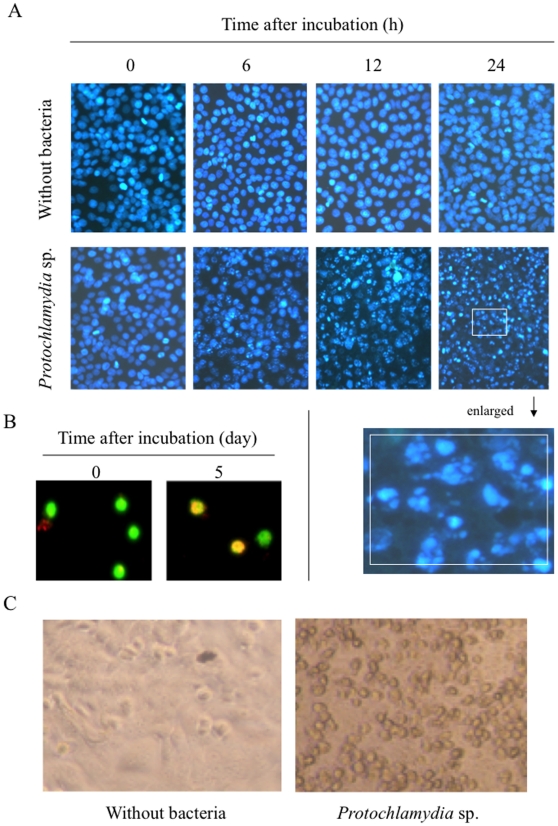
HEp-2 cell death induced by the addition of *Protochlamydia*. **A**) Representative images showing Time-course changes of cell death in HEp-2 cells after the addition of *Protochlamydia* (MOI 90). Cell death was estimated through the observation of nuclear morphological changes with DAPI staining under a fluorescent microscope. Enlarged images (white square) show typical morphological changes with blebbing and segmented nucleus indicating cell death. **B**) The ability of *Protochlamydia* to infect amoebae (*A. castellanii* C3). Infectious ability was estimated using FISH staining [24]. Green, eukaryotic 18S rRNA. Yellow, specific *Protochlamydia* signals. Magnification, ×100. **C**) Morphological changes of HEp-2 cells after the addition of *Protochlamydia* (MOI 90), estimated using light microscopy at 24 h after incubation. Cells in the presence of the bacteria exhibited morphological changes indicative of apoptosis, including rounding and detachment (See right panel).

We first assessed whether *Protochlamydia* could induce phenotypic alternation, such as cell death, in human immortal HEp-2 cells. HEp-2 cell morphology altered to include blebbing (Fig. 1A) and cellular detachment (Fig. 1C), after being incubated with the bacteria for 24 h. This indicated morphological traits of cell death, such as apoptosis. Cell viability was further confirmed using a trypan blue exclusion assay (data not shown). The prevalence of dead cells started to increase by approximately 20% at 6 h after incubation, and reached approximately 60–80% at 24 h after incubation. Since morphological changes peaked at MOI 90 (Fig. S1), the bacterial inoculation rate of this MOI was therefore used for the following experiments. The bacteria used for inoculations were observed to be viable as they were still capable of infecting the amoebae, which are natural host cells for *Protochlamydia* (Fig. 1B). These results strongly suggested that the cell death of HEp-2 cells induced by the inoculated *Protochlamydia* was apoptosis.

### Evaluation of the cell death as apoptosis

**Figure 2:**
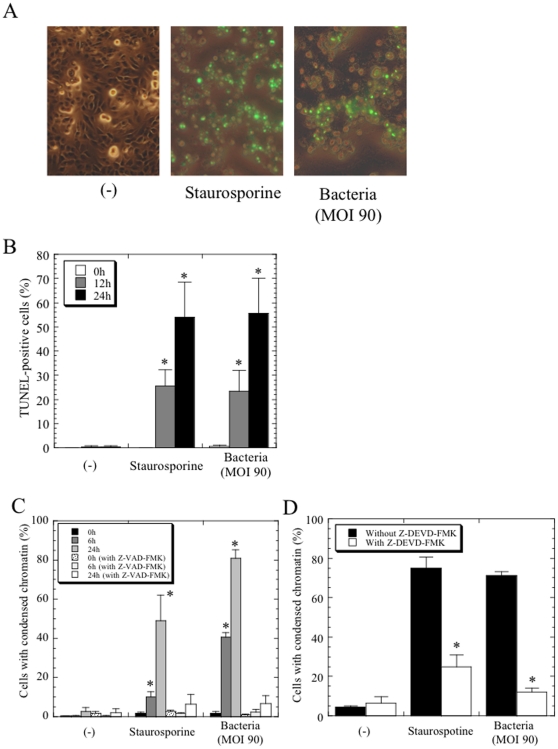
Cell death in HEp-2 cells induced by the addition of *Protochlamydia* is apoptosis. Apoptotic cell death after the addition of *Protochlamydia* (MOI 90) was estimated using the TUNEL assay (A, B) and DAPI staining (experiments with caspase inhibitors) (C, D). Staurosporine (10 µM) was used as a positive control to induce apoptosis. **A**) Representative images of apoptotic cells with phase contrast images at 24 h after incubation. Green, apoptotic cells. Magnification, ×100. **B**) The prevalence of apoptotic cells estimated by TUNEL assay with time-course changes. The percentage of apoptotic cells was measured under a microscope by counting at least 200 cells in three random fields for each culture sample. The data shown represent the means + standard deviations (error bars) (SD), obtained from at least three independent experiments performed in triplicate. *, *p*<0.05; significantly different from each data at immediately (0 h) after incubation. **C**) The prevalence of cells with condensed chromatin with time-cause changes in the presence or absence of the inhibitior, Z-VAD-FMK (general caspase inhibitor). The percentage of the cells was measured under a microscope by counting at least 200 cells in three random fields for each culture sample. The data shown represent the means + SD, obtained from at least three independent experiments performed in triplicate. *, *p*<0.05; significantly different from each data at immediately (0 h) after incubation. **D**) The prevalence of cells with condensed chromatin at 24 h after incubation in the presence or absence of the inhibitor, Z-DEVE-FMK (specific caspase-3 inhibitor). The data shown represent the means + SD, obtained from at least three independent experiments performed in triplicate. *, *p*<0.05; significantly different from each data without the treatment.

**Figure 3:**
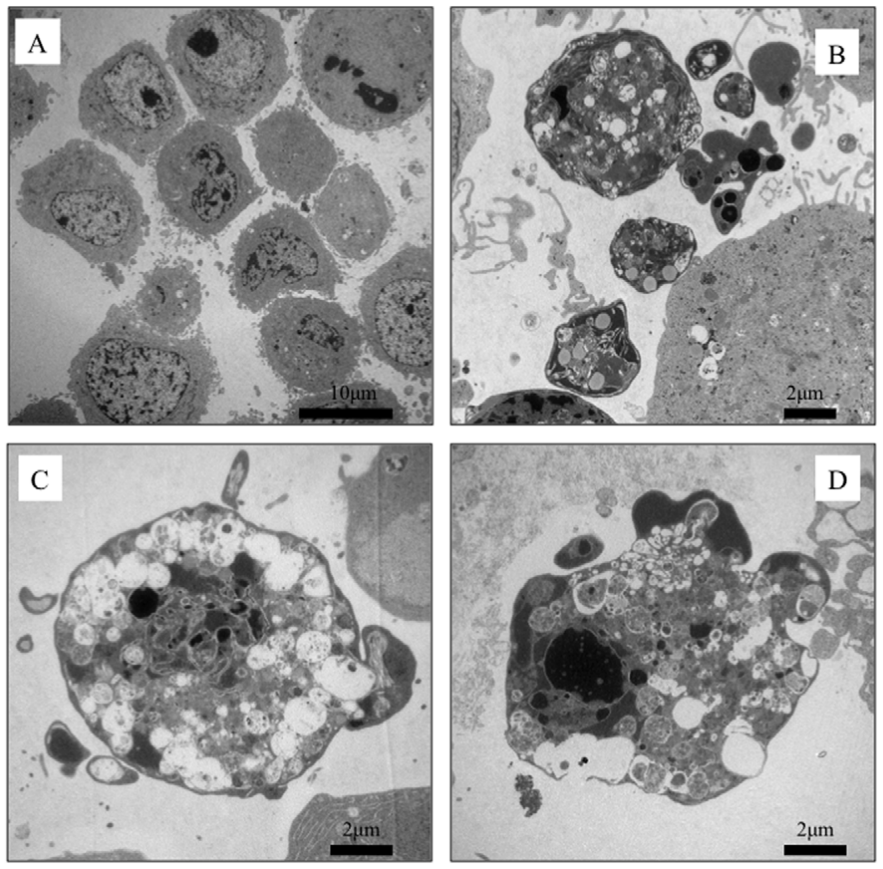
Representative TEM images of HEp-2 cells treated with *Protochlamydia* or staurosporine. Cells were cultured with bacteria adjusted at MOI 90 or staurosporine for 6 h, and then fixed. **A**) Negative control cells without any treatment. **B**) Cells treated with staurosporine (10 µM). **C and D**) Representative images of HEp-2 cells treated with the bacteria.

To assess the possibility of apoptotic cell death, we determined if the traits of this cell death were identical to the criteria of apoptosis using the TUNEL assay. Specific fluorescence spots on the cultured cells were observed in the presence of *Protochlamydia* and staurosporine (a positive control for apoptosis), demonstrating that the *Protochlamydia*-induced cell death was apoptosis (Fig. 2A). The prevalence of specific spots significantly increased with time over on the 24 h culture period (Fig. 2B). Similar morphological changes were also observed for the cells cultured with staurosporine, along with identical cell death rates as estimated using 4′,6-diamidino-2-phenylindole (DAPI) staining (Fig. 2B). It was also confirmed that the prevalence of dead cells was significantly inhibited by the treatment with a general caspase inhibitor Z-VAD-FMK, which is a cell-permeant pan caspase inhibitor (Fig. 2C) and a specific caspase-3 inhibitor Z-DEVD-FMK (Fig. 2D), respectively, suggesting that the cell deaths is unlikely to be pyroptosis without caspase-3 activation [14]. Although unusual in some features the transmission electron microscopy (TEM) morphology was consistent with cell death apoptosis (Fig. 3). We also confirmed that *Protochlamydia* could induce apoptosis in other mammalian immortal cells, including Vero, Jurkat and THP-1 cells (Fig. S2). Thus, these results indicated that the cell death on HEp-2 cells induced by the inoculation of *Protochlamydia* was apoptosis. Since apoptotic cells could easily be identified with DAPI staining, this staining method was used in all subsequent HEp-2 cell experiments to estimate the prevalence of apoptotic cells through the observation of morphological changes.

### Mechanism of apoptosis induced by the addition of *Protochlamydia*

**Figure 4:**
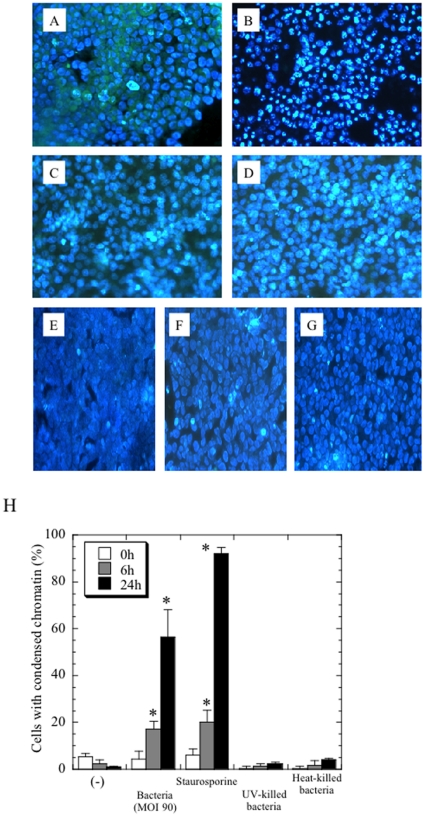
Viable *Protochlamydia* induced apoptosis. Cells were cultured with or without the bacteria adjusted at MOI 90 [or either heat- or UV-killed bacteria (equivalent MOI 90)] or other chlamydiae for 24 h, and then cell morphological changes were also estimated by DAPI staining. The representative images were captured at 24 h after incubation. Magnification, ×200. **A**) Negative control cells without any treatment. **B**) Cells treated with viable bacteria. **C**) Cells treated with heat-killed bacteria. **D**) Cells treated with UV-killed bacteria. **E, F and G**) Cells treated with other viable chlamydiae adjusted at MOI 90 [*C. trachomatis* D (E), *C. trachomatis* L2 (F) and *P. acanthamoebae* (G)]. **H**) Time-course changes in the prevalence of apoptotic cells. The data shown represent the means + SD, obtained from at least three independent experiments performed in triplicate. *, *p*<0.05; significantly different from each data immediately (0 h) after incubation.

We initially determined if either UV-treated or heat-treated bacteria could induce apoptotic cell death. Both bacterial treatments failed to induce cell death, indicating that viable bacteria are required for this phenomenon to occur (Fig. 4C, D and H). Whether the apoptosis is required for bacterial protein synthesis remains undetermined. In addition, inoculation with other viable chlamydiae [*Parachlamydia acanthamoebae* and *Chlamydia trachomatis* (serovars D and L2)] did not cause apoptotic cell death (Fig. 4 E–G), indicating that the observed apoptosis is specific to the *Protochlamydia* in the chlamydiae used for this study. In addition, because of short incubation period and observation at low magnification, we could not distinguish inclusion formed by *C. trachomatis* (D and L2) from cell nucleus.

**Figure 5:**
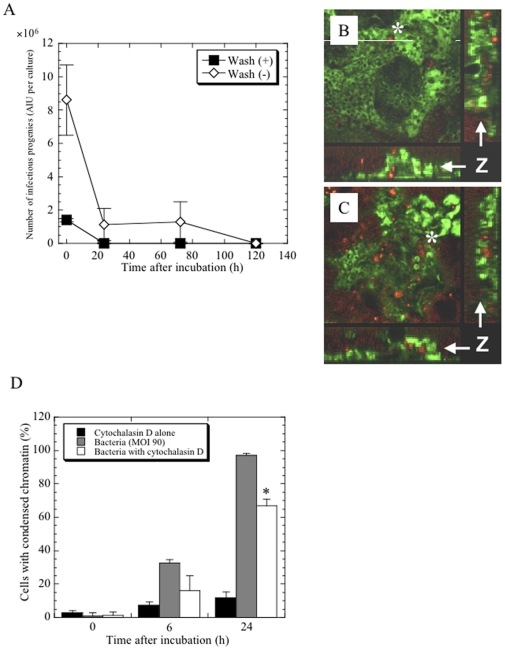
Changes of the number of bacterial infectious progenies within and without the cells, and effect of cytochalasin D on the induction of apoptosis. **A**) Number of bacterial infectious progenies in the cultures. After mixing with the bacteria, cells with or without washing were incubated for up to 5 days. The Bacterial numbers were estimated by the method described previously [26]. The data shown represent the means ± SD, obtained from at least three independent experiments performed in triplicate. See the Materials and Methods (*Cell cultures inoculated with bacteria*). **B**) **C**) Representative FISH images of surviving cells (B) and apoptotic cells (C) 5 days after incubation. Bacteria were rarely observed within the cells, regardless of apoptosis or not. Green indicates eukaryotic18S rRNA and yellow indicates specific *Protochlamydia* signals. Z, z-axis; *, specific signal of the bacteria detected inside the cells. **D**) Effect of cytochalacin D on the cell death with the bacteria. The data shown represent the means + SD, obtained from at least three independent experiments performed in triplicate. *, *p*<0.05; significantly different from bacteria alone at each time point.

We also assessed whether *Protochlamydia* could replicate and grow in cultured HEp-2 cells using the previously established AIU assay [26]. No increase in the number of bacterial infectious progenies was observed in the cultures, regardless of the treatment and washing to ensure bacterial attachment to the cells (Fig. 5A). This indicated that an increase of bacterial infectious progenies was not required for the induction of this apoptosis. Furthermore, although bacteria were not observed within the cells throughout the 24 h incubation (data not shown), at 5 days after incubation, the bacterial signal associated with fluorescent in situ hybridization (FISH) staining was barely observed inside the cells, regardless of apoptosis (Fig. 5B and C), suggesting that a role of bacterial uptake in the cell death induction may be minimal. However, the treatment with cytochalacin D, which is a critical inhibitor for pathogenic chlamydial invasion [27], [28], slightly inhibited the prevalence of dead cells (Fig. 5D), implying that the bacterial entry may be in part associated with this apoptosis. Further study should be needed to clarify this issue.

**Figure 6:**
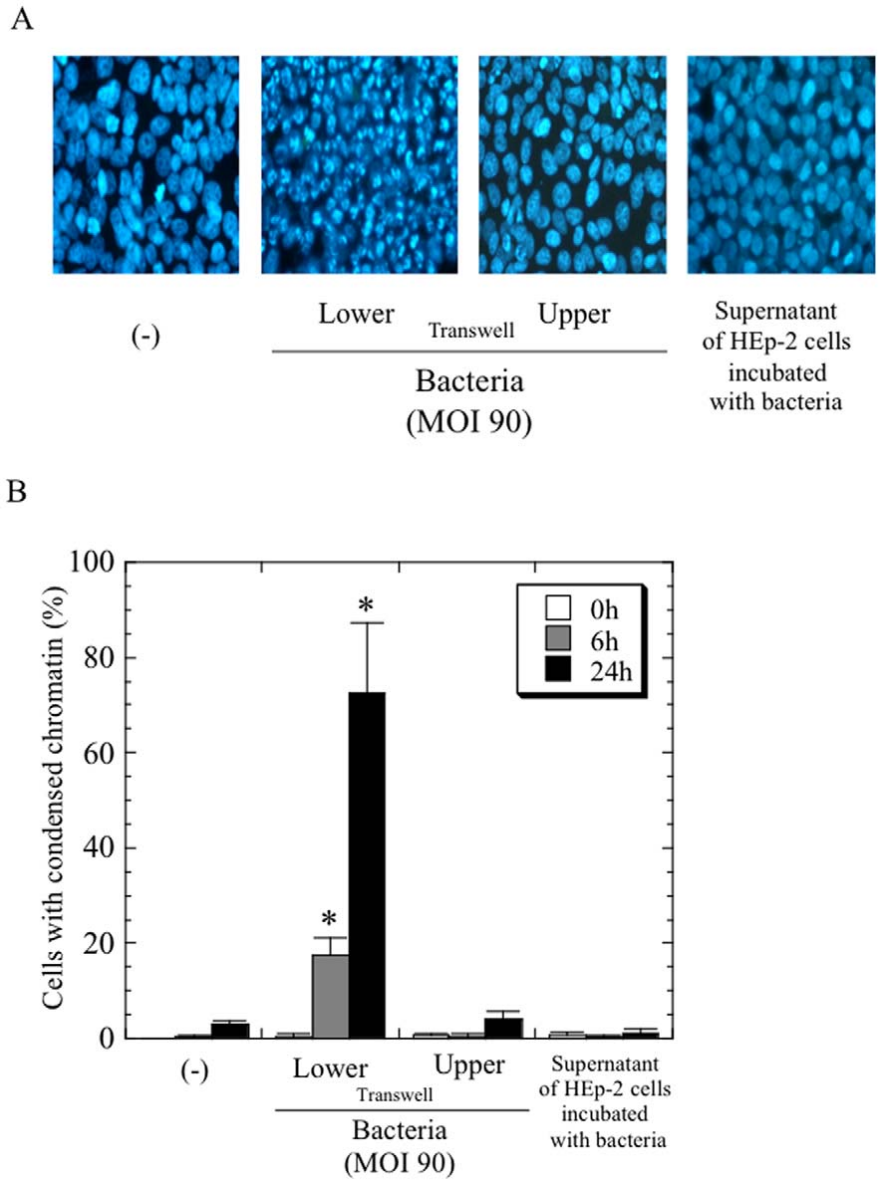
Induction of apoptosis requires the attachment of bacteria to HEp-2 cells. HEp-2 cells were cultured to both upper and lower chambers in Transwells. Cells in the upper chamber were subsequently inoculated with bacteria (MOI 90). **A**) Representative images of DAPI staining at 24 h after incubation. Magnification, ×200. **B**) Time-course changes of the percentage of apoptotic cells. The data shown represent the means + SD, obtained from at least three independent experiments performed in triplicate. *, *p*<0.05; significantly different from each data immediately (0 h) after incubation.

**Figure 7:**
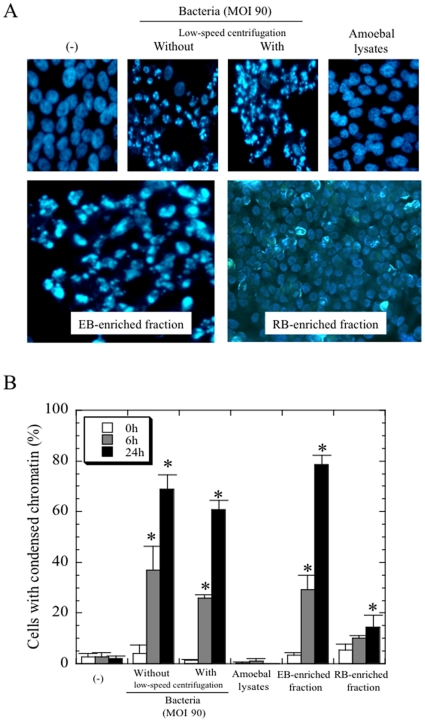
Enriched EB, but not RB or amoebal components, induced apoptosis in HEp-2 cells. **A**) Representative images showing that only EB (MOI 90), but not RB (equivalent MOI 90) or amoeba components, induced apoptosis at 24 h after incubation. Magnification, ×200. **B**) Time-course changes of the percentage of apoptotic cells. The data shown represent the means + SD, obtained from at least three independent experiments performed in triplicate. *, *p*<0.05; significantly different from each data immediately (0 h) after incubation.

Consequently, we assessed whether bacterial attachment to the cells is required for the observed cell death, by using a Transwell assay mounting membrane filter with a 0.45 µm-pore size, through which the bacteria could not pass. Apoptosis was induced in cells that were mixed with *Protochlamydia* in the lower space of the Transwell. In contrast, the cells cultured without *Protochlamydia* in upper space of Transwell did not develop any morphological changes indicative of apoptosis (Fig. 6). We also assessed the association of fluid factors secreted from either bacteria or cells with apoptosis. However, supernatants from HEp-2 cells with bacterial induced apoptosis did not produce any morphological changes on newly prepared HEp-2 cells (Fig. 6), and were similar to control cells without bacteria. Since the influence of a few amoebae cells contaminating the bacterial stock could not be ruled out, these amoebae were eliminated with low-speed centrifugation before being tested. Regardless of low-speed centrifugation, the prevalence of apoptosis in HEp-2 cells did not change, and moreover, the addition of amoebal lysate to the culture had no affect on morphological changes in the HEp-2 cells (Fig. 7). This demonstrated that there was no association between the amoebae and apoptosis.

We also assessed if either the EB or RB form is responsible for inducing apoptosis. The prevalence of apoptosis in HEp-2 cells mixed with EB was significantly higher than that with RB (Fig. 7). A slight increase in prevalence was observed when RB were added to cultured cells because of the effect of contamination with a few EB. Thus, we concluded that *Protochlamydia* EB, but not RB, could induce apoptosis. The attachment of viable bacteria to the cells, but not an increase of bacterial infectious progenies, is also required for the apoptosis.

### Effect of *Protochlamydia* on survival of primary cells, human PBMCs

**Figure 8:**
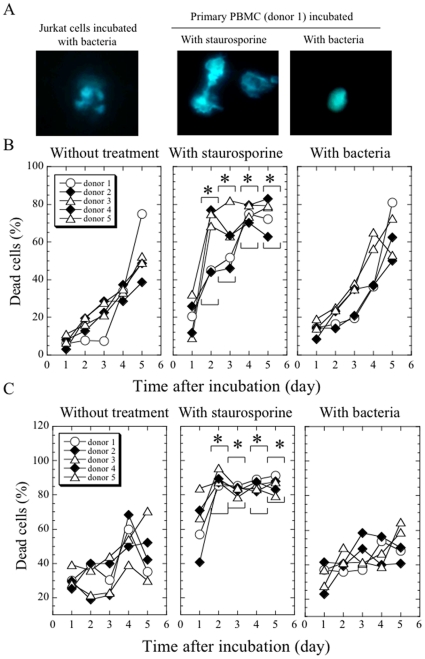
Cell death did not occur in PBMCs regardless of activation with PMA and ionomycin. **A**) Representative DAPI staining images showing that the bacteria induced cell death in immortal Jurkat cells, but not in primary PBMCs. Data was estimated at 24 h after incubation. Magnification, ×1,000. **B and C**) Time-course changes of the percentage of dead PBMCs with (C) or without (B) PMA and ionomycin. PBMC cell death prevalence was estimated using a trypan blue exclusion assay. The plot shown represents the mean value of the data from individual donors performed in duplicate. The averages in parentheses at each time point are compared. *, *p*<0.05; significantly different from each average for the “without treatment group” at each of the time points after incubation.

Because human immortal HEp-2 cells do not exhibit the traits of human primary cells, we further examined whether *Protochlamydia* could also induce cell death in primary human PBMCs with or without activation, which were prepared from the blood of five donors. Cell death did not occur in any of the PBMCs regardless of activation, although staurosporine clearly induced cell death in these cells [Fig. 8 (estimated by trypan blue exclusion assay), and Fig. S3 (estimated by DAPI staining)].

## Discussion

It is increasingly evident that the biological and genomic traits of *Protochlamydia* are unique when compared with other bacterial pathogens such as the pathogenic chlamydiae. In particular, the *Protochlamydia* UWE25 genome was reported by Horn *et al*., [10] to be approximately twice as large as the genomes of any other pathogenic chlamydiae investigated, including *C. trachomatis* or *C. pneumoniae*. In their study, only a few pseudogenes and genome remnants were detected, which were mostly associated with the transposase gene. This indicated that the genome of *Protochlamydia* is not in the process of becoming smaller but has stabilized at approximately 2.4 Mb [10]. In contrast, pathogenic chlamydiae have a reduced genomic size because they inhabit in a more homeostatic niche than that of *Protochlamydia*, which is exposed to fluctuating environmental conditions in its amoebal host [10]. Therefore, *Protochlamydia* is likely to have retained many functional molecules, which the pathogenic chlamydiae have lost as a consequence of adapting to a stable host environment. Therefore, the potential of *Protochlamydia* to induce apoptosis within human immortal cells is of interest, as it offers new insights into host-parasite relationships, and moreover, contributes to the development of a novel strategy for removing cells persistent infected with pathogens, through activation of the cells encountered unknown proteins that still continued to be retained in *Protochlamydia*, but has been lost in pathogenic chlamydiae.

While further study is needed to determine the mechanism by which *Protochlamydia* induces apoptosis in human immortal HEp-2 cells, it is clear that the attachment of viable bacteria is necessary and that an increase of bacterial infectious progenies within cells is not required. Genome sequence data for *Protochlamydia* UWE25 suggests no association of the LPS endotoxin with cell death, because it lacks the gene relating to LPS synthesis [10]. Dead bacteria completely lost the ability to induce apoptosis, indicating that bacteria with completely lost metabolic activities are incapable of inducing apoptosis. Although *Protochlamydia* survival under extracellular conditions requires further investigation, support for our results is provided by recent work showing the long-term extracellular activity of chlamydial EB [29], [30].

While the mechanism by which *Protochlamydia* induces apoptosis is currently unknown, it is possible that dedicated effectors molecules secreted by the bacterial cytoplasm might contribute to the observed cell death. Previous research of the bacterial genome indicates the conservation of two types of secretion machines, type III and type IV [31], although the associated effectors molecules have yet to be characterized. It is well known that *C. trachomatis* injects a variety of proteins into its host cells to ensure the survival of this pathogen. Among them, inclusion membrane proteins (Incs) are of particular interest. Even though up to 70–90 Incs proteins are predicted for chlamydial genomes, only a few of these proteins have been characterized beyond their localization to the inclusion membrane [32], [33], [34], [35]. A recent study demonstrated that *Protochlamydia* UWE25 possessed multiple functional Incs transferred on inclusion membrane, which might be responsible for fusion of chlamydial inclusions and host cellular vesicles containing lipid and metabolites [36]. Since known Inc proteins do not share a sequence homology, it is possible that some unknown proteins have a detrimental effect on host cells, such as apoptosis.

Recent proteomic analysis has proposed a model of the *P. amoebophila* outer membrane, indicating the presence of considerably high amounts of putative lipoproteins [37]. Such bacterial lipoproteins have a wide variety of functions and can activate several human cell types; for example, mycoplasma lipoprotein can activate cellular immune response via toll-like receptor, followed by NF-κB activation with cross-linkage leading to cell death [38], [39]. Therefore, it is also possible that lipoproteins in the outer membrane are associated with apoptosis, although their active translocation mechanism is currently unknown.

Previous research indicated that *P. acanthamoebae*, a member of the environmental chlamydiae, could induce apoptosis in human macrophages [20]. However, in our study *P. acanthamoebae* did not induce apoptosis in HEp-2 immortal cells. Such disparity may be associated with differences in culture conditions or cell line characteristics. In addition, individual environmental chlamydiae species have evolved into amoebae living into domestic area, implying that speed of evolution occurred into some bacteria differs from the others, regardless of same species. Therefore, it could not deny that intraspecific variations within certain environmental chlamydiae might also reflect the difference in results.

Although the reasons as to why PBMCs did not succumb to bacterial induced apoptosis remain unclear, it is easily thought that intracellular activation against *Protochlamydia* in immortal cells may differ from that in primary PBMCs; in fact it is well known that immortal cells such as cancer cells display increased proliferation with altered homeostasis via changes of various cellular functions as compared with primary cells. Based upon these, we would like to propose the following explanation: Immortal cells have evolved the ability to undergo rapid genome replication, regardless of limited *in vitro* culture conditions, when compared with normal primary cells that have been freshly collected from tissues or blood. It is therefore possible that a distance gap point of view from the bacteria is much bigger to immortal cells than that to primary cells, implying that the treatment with *Protochlamydia* could selectively cause cell death on cancer cells. Further study is needed to verify this idea, which can potentially be used in the development a novel drug that is specific and effective against cancer cells.

In conclusion, for the first reported time we have demonstrated that the amoebal endosymbiont *Protochlamydia* can induce apoptosis in a human-immortal HEp-2 cell line, but not in PBMCs. Thus, this endosymbiont has potential as a useful biological tool for elucidating novel host-parasite relationships.

## Methods

### Bacteria stocks and assessment of bacterial numbers

*Protochlamydia* used in this study was identified as an environmental amoebae endosymbiont [16SrRNA sequence: AB506679 (99.2% identities against *Protochlamydia* UWE25 16SrRNA sequence), and were maintained within infected R18 amoebae (genotype T4), found in environmental *Acanthamoeba* strains isolated from a river of Sapporo city, Japan [24], [25]. Briefly, the infected cells were harvested and disrupted by freeze thawing. After centrifugation at 180× g for 5 min to remove cell debris, bacteria were concentrated by high-speed centrifugation at 800× g for 30 min. The bacterial pellet was resuspended in sucrose-phosphate-glutamic acid buffer containing 0.2 M sucrose, 3.8 mM KH_2_PO_4_, 6.7 mM Na_2_HPO_4_ and 5 mM L-glutamic acid (pH 7.4), and then stored at −80°C until needed. Killed bacteria were prepared by UV exposure (15 W, 5 cm, 3 h) and heat treatment (95°C, 30 min). EB-enriched and RB-enriched fractions were also obtained from the bacterial stocks using ultra-high speed centrifugation with percoll (Sigma, St. Louis, MO) density gradient at 10,000× g for 1 h [40]. The number of infective *Protochlamydia* progeny (EB) was determined with an amoeba-infectious unit (AIU) assay, using a co-culture of the amoebae, established previously [26]. Briefly, each sample containing viable *Protochlamydia* was serially diluted from 100–10^−7^ with PYG broth and incubated with *A. castellanii* C3 (see below “*Amoebae and human cells*”) (10^4^ or 10^5^ per well) in PYG broth with or without cycloheximide (200 µg/ml) in 96-well plates for 2 days. The infection rate of *Protochlamydia* to amoebae (amoeba-infectious dose, AID) in each well was determined by microscopy at a magnification of 100×, following DAPI staining. Ten fields were randomly selected for this assessment. The AIDs for a sample were plotted as a logistic sigmoid dilution curve using statistical software (KaleidaGraph 3.6; Hulinks, Tokyo, Japan). The formula logically draws a specific sigmoid curve via statistical software and shows a dilution rate corresponding to the mid-value of the amoeba-infectious rate (AID_50_). Finally, the viable bacterial numbers in cultures, defined as AIU, can be determined based on the value of AID_50_. The number of RB was adjusted under a chtoatometer. To assess the influence of amoebal components or whole amoeba on apoptosis, amoebal lysates prepared from C3 amoebae (approximately 10^6^ cells) by freeze-thawing and the bacterial solution, from which amoebae were eliminated by low-speed centrifugation (200×g, 10 min), were also used for this study. *Parachlamydia acanthamoebae* [Bn9 (ATCC VR-1476)] was purchased from American Type Culture Collection (Manassas, VA). The bacteria were propagated in the amoeba cell culture system in the same way of *Protochlamydia*. Also, *C. trachomatis* 434/Bu (LGV: serovar L2) and UX-7 (serovar D) were purchased from ATCC. Bacteria were propagated in a HEp-2 cell culture system as described previously [29].

### Amoebae and human cells

Free-living amoebae, *A. castellanii* C3 (ATCC 50739), was purchased from the American Type Culture Collection, and used to assess the bacterial infectious progenies by AIU assay. As mentioned above, R18 amoebae harboring endosymbiotic *Protochlamydia* that were isolated from a natural environment were also used to prepare bacterial stocks. Both amoebae were maintained in PYG broth [0.75% (w/v) peptone, 0.75% (w/v) yeast extract and 1.5% (w/v) glucose] at 30°C [26]. Immortal cells [Epithelial cell line cells (HEp-2, Vero cells), immune cells (THP-1, Jurkat cells)] and primary PBMCs prepared from whole blood, provided by healthy volunteers, using density gradient centrifugation with Histopaque (Sigma) were used for assessment of cell death. Informed consent with written was obtained from all volunteers in this study, and the study was approved by the ethics committee of Faculty of Health Sciences, Hokkaido University.

### Cell cultures inoculated with bacteria

Immortal cells (2×10^5^ cells) and PBMCs (5×10^5^ cells) were cultured with or without bacteria adjusted at MOI 10–100 or with staurosporine (10 µM) (Sigma) as a positive control for the induction of apoptosis, for up to 24 h (for HEp-2 cells) or 5 days (for PBMCs) at 37°C in 5% CO_2_ in DMEM (for HEp-2, Vero) or RPMI medium (for THP-1, Jurkat, PBMCs), containing 10% heat-inactivated fetal calf serum. Immortal cells and PBMCs were also cultured in the presence or absence of the bacteria with or without Z-VAD-FMK (100 µM) (general caspase inhibitor) (Promega, Madison, WI), Z-DEVE-FMK (specific caspase-3 inhibitor) (100 µM) (R&D Systems, Minneapolis, MN), or cytochalasin D (1 µM) (Sigma). In some experiment, HEp-2 cells were cultured in a Transwell (pore size, 0.45 µm), to prevent the direct attachment of bacteria. PBMCs activated with phorbol 12-myristate 13-acetate (PMA) (25 ng/ml) (Sigma) and ionomycin (1 µg/ml) (Sigma) were also used for this study. No cytotoxicity of these drugs at working concentration in the cells was confirmed.

### Assessment of cell death

Phenotypic alternation of the human cells was assessed using DAPI staining, TEM and the TUNEL assay, according to the classification method previously described by Kroemer *et al* [14]. Cell death of PBMCs was also estimated using a trypan blue exclusion assay, in addition to assessing changes of nuclear morphology by DAPI staining. TUNEL assays were performed using a commercial *in situ* apoptosis detection kit (Takara, Shiga, Japan), according to the manufacturer's protocol. Morphological analysis with TEM was performed according to the previously described method [16]. In brief, bacteria within cultures were immersed in a fixative containing 3% glutaraldehyde in 0.1 M PBS pH 7.4, for 24 h at 4°C. After a brief wash with PBS, bacteria were processed for alcohol dehydration and embedded in Epon 812. Ultrathin sections of the cells were stained with lead citrate and uranium acetate before viewing by TEM (Hitachi H7100; Hitachi, Tokyo, Japan).

### Statistical analysis

Comparison of bacterial numbers in the *in vitro* experiment was assessed using an unpaired *t*-test. A *p*-value of less than 0.05 was considered significant.

## Supporting Information

Figure S1 **Representative images (A) and numbers of dead cells (B) in HEp-2 cell cultures induced by the addition of** ***Protochlamydia*** **dependent upon MOI.** Cells were cultured with or without the bacteria adjusted at MOI 10–100 for up to 24 h. The number of dead cells was estimated using DAPI staining. The data shown represent the means + SD, obtained from at least three independent experiments performed in triplicate. *, *p*<0.05; significantly different from each data for the “without bacteria”.
(TIF)

Figure S2 **Representative images (A) and numbers of dead cells (B) in either Vero, THP-1, or Jurkat cells, induced by the addition of** ***Protochlamydia*** **.** Cells were cultured with or without bacteria (MOI 90) or staurosporine for up to 24 h. The data shown represent the means + SD, obtained from at least three independent experiments performed in triplicate. *, *p*<0.05; significantly different from each data for the “(-)” at immediately (0 h) after incubation.
(TIF)

Figure S3 **Representative images of cells with condensed chromatin (A) and the prevalence of dead cells in PBMC cultures prepared from three donors (donor 1–3; See the** **Fig. 8** **) in the presence or absence of the bacteria (MOI 90) or staurosporine, with or without PMA and inomycin for up to 3 days.** **A**) Representative DAPI staining images showing that the bacteria induced cell death in PBMCs only limited with staurosporine. The data were estimated at 24 h after incubation. Magnification, ×1,000. **B**) The percentage of dead cells in PBMCs. The prevalence of dead cells was estimated using DAPI staining. The data shown represent the means + SD, obtained from at least three independent experiments performed in triplicate. *, *p*<0.05; significantly different from each data for the “(-)” at each time point.
(TIF)
