# Supplementary figures and images for: Amoebal Endosymbiont Protochlamydia Induces Apoptosis to Human Immortal HEp-2 Cells
Source: PLoS One. 2012 Jan 19;7(1):e30270. doi: 10.1371/journal.pone.0030270 (PMC3261889; doi:10.1371/journal.pone.0030270)

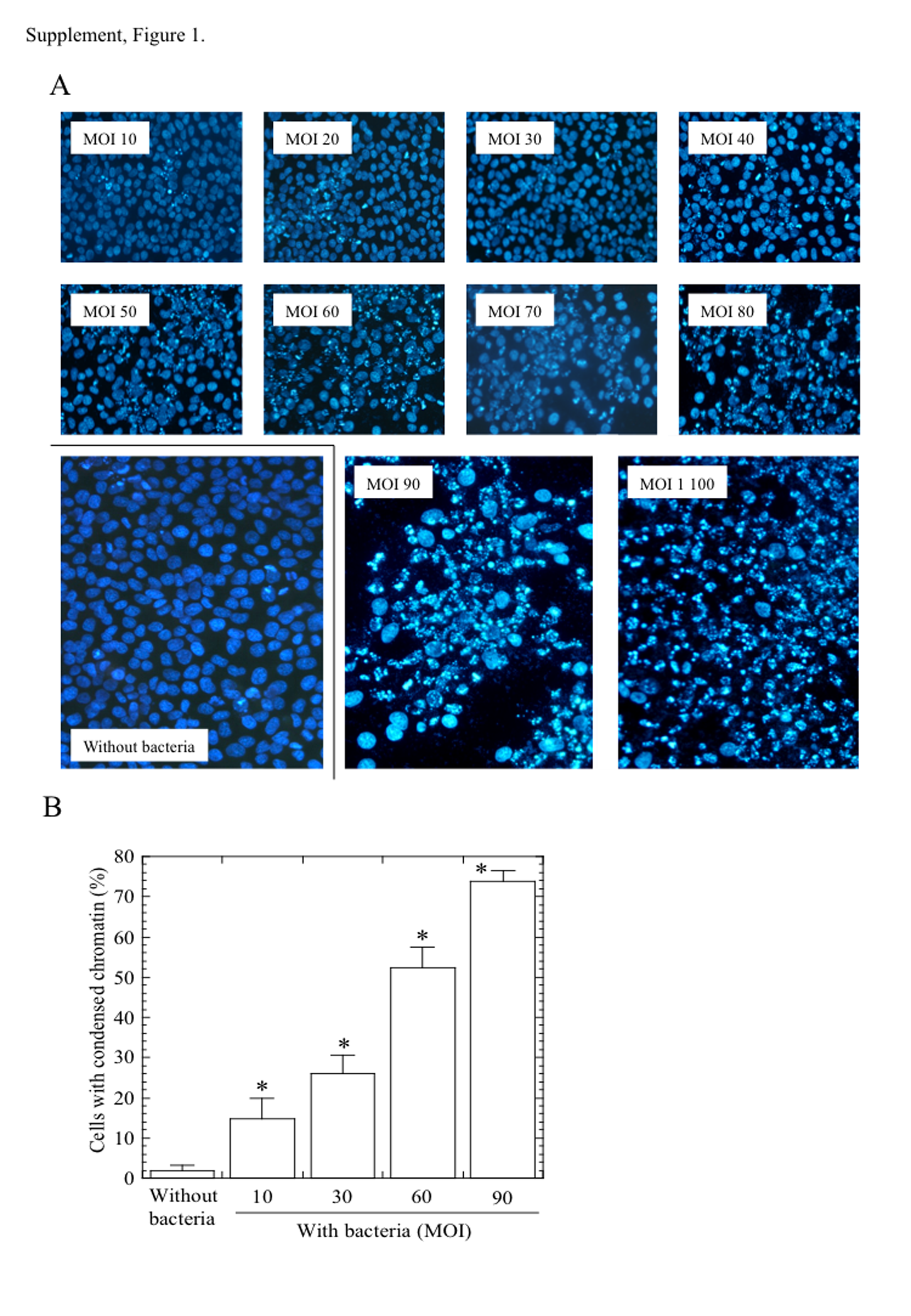

Supplement: Figure S1 — Representative images (A) and numbers of dead cells (B) in HEp-2 cell cultures induced by the addition of Protochlamydia dependent upon MOI. Cells were cultured with or without the bacteria adjusted at MOI 10–100 for up to 24 h. The number of dead cells was estimated using DAPI staining. The data shown represent the means + SD, obtained from at least three independent experiments performed in triplicate. *, p<0.05; significantly different from each data for the “without bacteria”. (TIF) [file pone.0030270.s001.tif]

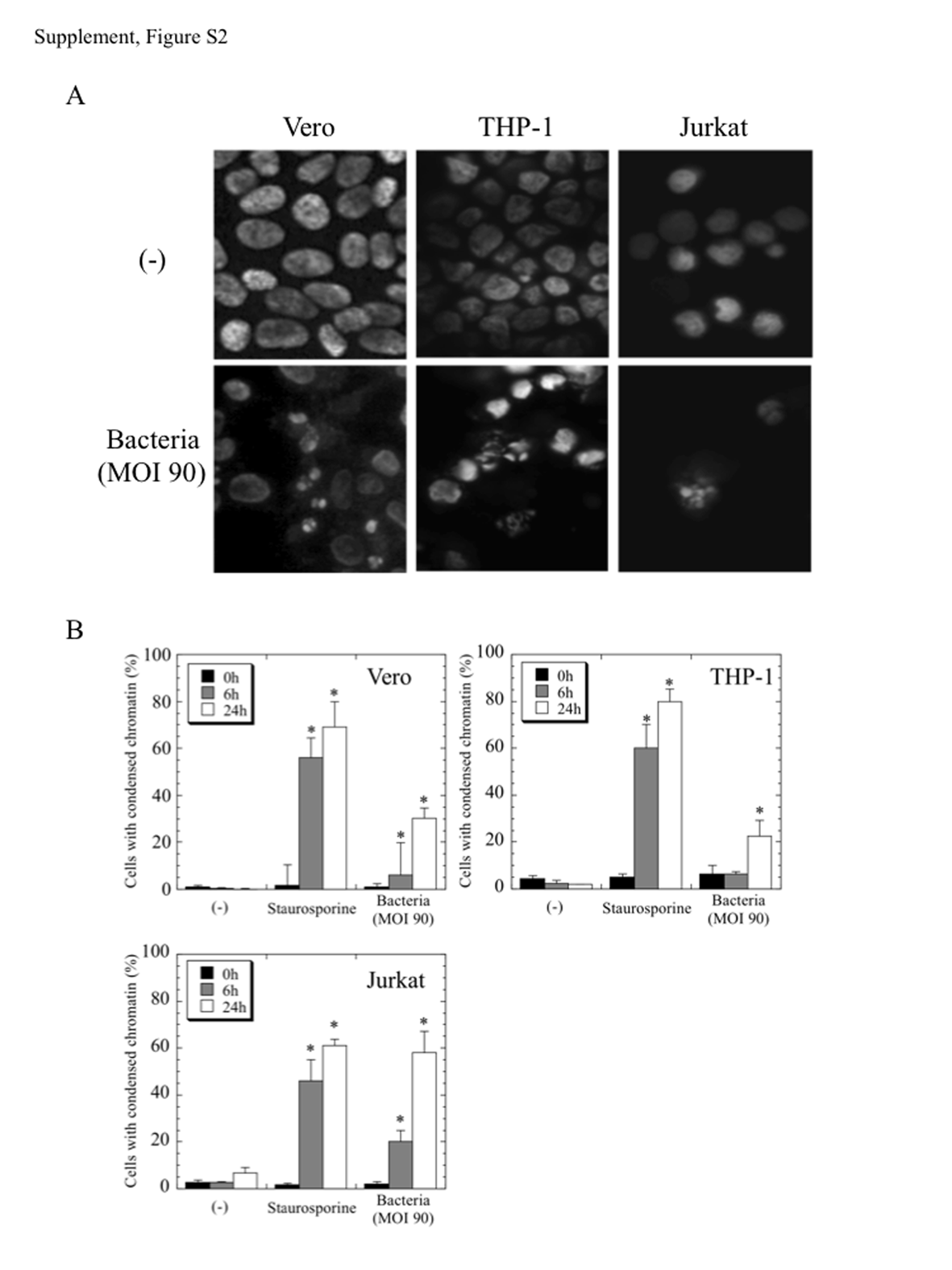

Supplement: Figure S2 — Representative images (A) and numbers of dead cells (B) in either Vero, THP-1, or Jurkat cells, induced by the addition of Protochlamydia . Cells were cultured with or without bacteria (MOI 90) or staurosporine for up to 24 h. The data shown represent the means + SD, obtained from at least three independent experiments performed in triplicate. *, p<0.05; significantly different from each data for the “(-)” at immediately (0 h) after incubation. (TIF) [file pone.0030270.s002.tif]

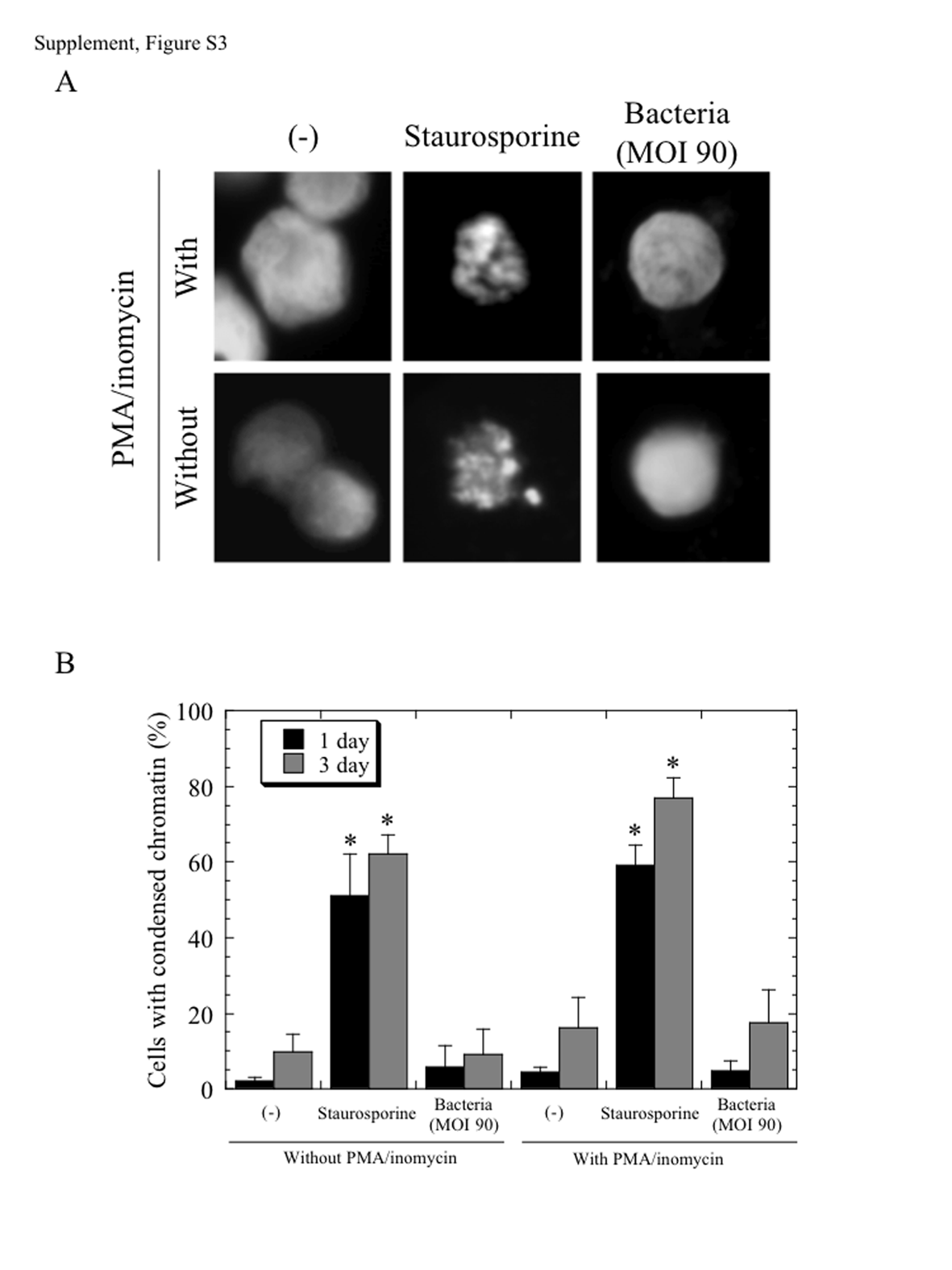

Supplement: Figure S3 — Representative images of cells with condensed chromatin (A) and the prevalence of dead cells in PBMC cultures prepared from three donors (donor 1–3; See the Fig. 8 ) in the presence or absence of the bacteria (MOI 90) or staurosporine, with or without PMA and inomycin for up to 3 days. A) Representative DAPI staining images showing that the bacteria induced cell death in PBMCs only limited with staurosporine. The data were estimated at 24 h after incubation. Magnification, ×1,000. B) The percentage of dead cells in PBMCs. The prevalence of dead cells was estimated using DAPI staining. The data shown represent the means + SD, obtained from at least three independent experiments performed in triplicate. *, p<0.05; significantly different from each data for the “(-)” at each time point. (TIF) [file pone.0030270.s003.tif]
